# Molecular Simulation and Statistical Learning Methods toward Predicting Drug–Polymer Amorphous Solid Dispersion Miscibility, Stability, and Formulation Design

**DOI:** 10.3390/molecules26010182

**Published:** 2021-01-01

**Authors:** Daniel M. Walden, Yogesh Bundey, Aditya Jagarapu, Victor Antontsev, Kaushik Chakravarty, Jyotika Varshney

**Affiliations:** VeriSIM Life Inc., 1 Sansome St, Suite 3500, San Francisco, CA 94104, USA; daniel.walden@verisimlife.com (D.M.W.); yogesh.bundey@verisimlife.com (Y.B.); aditya.jagarapu@verisimlife.com (A.J.); victor.antontsev@verisimlife.com (V.A.); kaushik.chakravarty@verisimlife.com (K.C.)

**Keywords:** solubility, bioavailability, drug development, amorphous solid dispersions, molecular modeling, molecular dynamics, machine learning

## Abstract

Amorphous solid dispersions (ASDs) have emerged as widespread formulations for drug delivery of poorly soluble active pharmaceutical ingredients (APIs). Predicting the API solubility with various carriers in the API–carrier mixture and the principal API–carrier non-bonding interactions are critical factors for rational drug development and formulation decisions. Experimental determination of these interactions, solubility, and dissolution mechanisms is time-consuming, costly, and reliant on trial and error. To that end, molecular modeling has been applied to simulate ASD properties and mechanisms. Quantum mechanical methods elucidate the strength of API–carrier non-bonding interactions, while molecular dynamics simulations model and predict ASD physical stability, solubility, and dissolution mechanisms. Statistical learning models have been recently applied to the prediction of a variety of drug formulation properties and show immense potential for continued application in the understanding and prediction of ASD solubility. Continued theoretical progress and computational applications will accelerate lead compound development before clinical trials. This article reviews in silico research for the rational formulation design of low-solubility drugs. Pertinent theoretical groundwork is presented, modeling applications and limitations are discussed, and the prospective clinical benefits of accelerated ASD formulation are envisioned.

## 1. Introduction

Drug solubility plays a vital role in the drug discovery and development pipeline, with around 40 to 70% of drugs displaying poor aqueous solubility due to highly stable solid lattice arrangements and/or pronounced hydrophobicity [1,2,3]. Decreases in bioavailability due to poor solubility are prevalent for oral dosages in which therapeutic compounds must be in solution to reach systemic circulation and achieve clinically relevant concentrations at the site of action. Considerable attention during discovery and lead optimization is therefore given to compound structural characteristics and the resulting physicochemical properties that affect solubility [4,5], with notable parameters, among many others, being a drug’s molecular weight, octanol:water partition coefficient (log*P*) [6], and possible acid-base ionization equilibria (p*K*_a_) [7]. There is complex interplay and tradeoff between the optimal chemical scaffold and functional groups for physiological response at the target and the ensuing effect on solubility and oral bioavailability [8]. Overcoming low drug solubility while maintaining critical molecular components for therapeutic activity often necessitates precise formulation strategies during the development stage [9,10,11].

**Figure 1:**
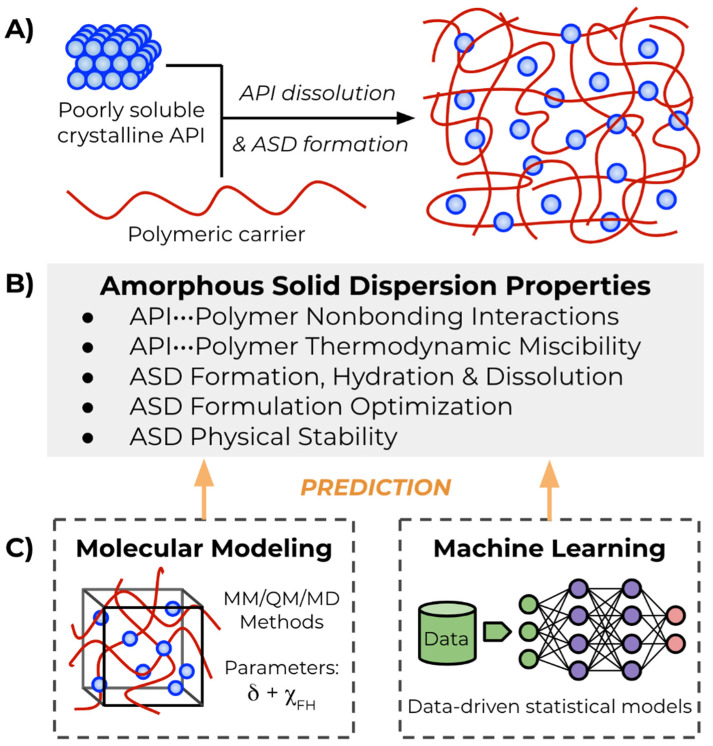
(**A**) Forming an ASD (amorphous solid dispersion) from mixing poorly water-soluble APIs (active pharmaceutical ingredients) with polymeric carriers. (**B**) General ASD properties and phenomena covered in this review. (**C**) Molecular modeling and machine learning are in silico methods for predicting ASD properties and phenomena. Miscibility estimated by calculation of solubility (*δ*) and Flory–Huggins interaction (χ_FH_) parameters.

A number of approaches exist for addressing the poor solubility of BCS (Biopharmaceutics Classification System) class II (low solubility, high permeability) and IV (low solubility, low permeability) active pharmaceutical ingredients (APIs) [1], including particle size reduction (micronization) [12,13], salt formation and co-crystallization [14,15], lipid-based delivery [16,17], and cyclodextrin complexation [18]. Among the variety of strategies, amorphous solid dispersions (ASDs) have seen consistent use as formulation and drug delivery systems, especially for BCS class II APIs [19,20,21,22]. In this system, lipophilic API is dispersed with a carrier compound, often a water-soluble amphiphilic polymer excipient, forming a multi-component matrix (Figure 1A). Compared to the more thermodynamically stable API crystalline form, once dispersed within the carrier matrix, the API occupies an elevated energetic state, and solubility and dissolution rate enhancement of the mixed ASD phase relative to crystalline API results from increased solvent-exposed surface area [23]. While this instability is beneficial due to the increased dissolution rate, relative ASD instability incurs a practical challenge of crystallization out of the amorphous state during shelf life [24]. An ideal ASD must strike a delicate balance between sufficient API saturation within the carrier and recrystallization [25]. Predicting the stability and miscibility of a given API within a carrier and the resulting solubility and dissolution rate of the mixture (Figure 1B) is of paramount importance to guide pharmaceutical formulation and production decisions. 

Several experimental methods have been developed and applied towards understanding and characterizing ASD solid-state nanostructure and properties [26,27]. Key solid-state endeavors include probing for regions of API crystallinity and the presence of impurities, both of which can serve as further crystal nucleation sites [28]. Measurement of the glass transition temperature (*T*_g_) routinely guides API–carrier miscibility, homogeneity, and physical stability evaluations [29]. Experimental deduction of intermolecular interactions occurring between API and carrier yields fundamental indicators of miscibility and overall ASD stability [30]. While experimental methods have yielded considerable understanding of the nanostructure and solid-state properties of ASD formulations, the metastable ASD system can also provide a significant challenge for experimental characterization. Appropriately matching a given excipient carrier to an API remains a chance approach in terms of concurrently optimizing API–carrier miscibility, ASD rate of drug release, and long-term ASD storage stability. The difficulty of rational excipient screening and lengthened development cycles due to ASD characterization experiments both contribute to the relatively small number of drugs prepared as solid dispersions ultimately reaching the market, as shown in Table 1 [31,32].

Computational approaches allow further understanding of ASD phenomena, especially when working in tandem to complement and inform experiments, and to assist in the development of predictive models for rational formulation design (Figure 1C). Molecular modeling and simulation techniques assist in elucidating important stabilizing intermolecular interactions between API and carrier, predict solubility parameters, simulate ASD formation and dissolution mechanisms, and generate descriptors for quantitative structure–property relationships (QSPR) [33,34]. Furthermore, machine learning methods are only just recently showing prominent applications to solid dispersion modeling and prediction. In this review, recent examples of molecular and statistical modeling and prediction as applied to the study of ASD intermolecular interactions, miscibility, formation, and stability are highlighted.

## 2. Theoretical Background

Two widely applied theoretical approaches for predicting the solubility of solid dispersions are the Hildebrand and Hansen solubility parameters and the Flory–Huggins (FH) interaction parameter [35,36,37,38,39]. Both models were originally developed to probe thermodynamic solute–solvent miscibility. In the context of API–polymer carrier miscibility, the theories are extended for polymer carrier systems by assuming the polymer as the solvent and the API as solute in a mixed system. The principle ultimately governing favorable mixing is the Gibbs free energy change upon mixing, given by Equation (1):

(1) $$\Delta G_{\mathrm{mix}}=\Delta H_{\mathrm{mix}}-T\Delta S_{\mathrm{mix}}$$

where *T* is the absolute temperature, and ∆*G*_mix_, ∆*H*_mix_, and ∆*S*_mix_ represent the change in free energy, enthalpy, and entropy between the unmixed and mixed components at constant pressure.

### 2.1. Solubility Parameters, δ

The Hildebrand solubility parameter, *δ*, is given as the square root of the cohesive energy density (*CED*) [38,40], defined as the per volume energy required to break intermolecular bonds and vaporize one mole of molecules from the liquid phase (Equation (2)):

(2) $$\delta =\sqrt{CED}=\sqrt{\frac{E_{\mathrm{vap}}}{V}}=\sqrt{\frac{\Delta H_{\mathrm{vap}}-\;RT}{V}}$$

where *E*_vap_ is the energy of vaporization, ∆*H*_vap_ is the change in the enthalpy of vaporization associated with the phase change, *V* is the molar volume of the liquid at the temperature of vaporization, *R* is the ideal gas constant, and *T* is the absolute temperature. Since the cohesive energy density is proportional to the enthalpy of vaporization, the magnitude of *δ* gives an indication of the strength of the intermolecular forces in the condensed state. The change in enthalpy upon mixing can be defined in terms of solubility parameters, as shown in Equation (3):

(3) $$\Delta H_{\mathrm{mix}}=V_{t}{\left(\delta_{1}-\delta_{2}\right)}^{2}\Phi_{1}\Phi_{2}$$

where *V*_t_ is the total volume of the mixture, *δ* and Φ are the solubility parameters and volume fractions of the individual mixture components, respectively. The entropic contribution to the free energy of mixing, ∆*S*_mix_, is positive as more ordered states disperse during mixing. Hence, favorable mixing occurs, as indicated by a negative ∆*G*_mix_, when the individual solubility parameters of the mixture components (*δ*_1_ and *δ*_2_) are close in value and ∆*H*_mix_ approaches zero. As applied to API–polymer carrier systems, a heuristic for estimating miscibility based on differences between component solubility parameters indicates ∆*δ*_API-polymer_ < 7 MPa^1/2^ as miscible and ∆*δ*_API-polymer_ > 10 MPa^1/2^ as immiscible, and values in between the thresholds as borderline miscible [37].

Hansen extended the Hildebrand solubility parameter to a three-dimensional space based on intermolecular attractive forces in order to more accurately model solutions containing polar compounds, as Hildebrand’s initial theory was developed based on hydrocarbon solvents [39]. In Hansen space, the solubility parameter is decomposed into a sum of contributions from three non-bonding interactions, as shown in Equation (4):

(4) $$\delta^{2}=\delta_{h}^{2}+\delta_{p}^{2}+\delta_{d}^{2}$$

where *δ*_h_, *δ*_p_, and *δ*_d_ are the hydrogen-bonding, polar, and dispersion contributions, respectively.

Group contribution methods calculate solubility parameters from molecular structure and can provide a rapid method for miscibility estimation if the structural fragment constants are available for all components of the compound of interest [41]. Two commonly applied group contribution methods include that of Fedors [42] and the Hoftyzer-Van Krevelen method [43], the latter shown below (Equation (5)) for estimating the Hansen solubility parameter:

(5) $$\delta_{h}=\sqrt{\frac{\sum^{\;}E_{\mathrm{hi}}}{V}},\;\;\;\;\;\delta_{p}=\sqrt{\frac{\sum^{\;}F_{\mathrm{pi}}^{2}}{V}},\;\;\;\;\;\delta_{p}=\frac{\sum^{\;}F_{\mathrm{di}}}{V}$$

where *E*_hi_, *F*_pi_, and *F*_di_ are the molar group attraction constants due to hydrogen-bonding, polar, and dispersion components, respectively, and *V* is the molar volume.

### 2.2. Flory–Huggins (FH) Interaction Parameter, χ

Another common theory for estimating miscibility between API and polymers is Flory–Huggins theory. One disadvantage of solubility parameters addressed by FH theory is the entropic contribution to ∆*G*_mix_, which potentially becomes important to incorporate due to the structural complexity and flexibility of polymeric carriers as the mixture deviates from an ideal solution [36]. In FH theory, the API–polymer system is modeled as a mixture where the average energy of interaction between each component depends on the FH interaction parameter, χ, and is related to the free energy change upon mixing via Equation (6) [11]:

(6) $$\frac{\Delta G_{\mathrm{mix}}}{RT}=\left(\frac{\Phi_{1}}{r_{1}}\right)ln\Phi_{1}+\left(\frac{\Phi_{2}}{r_{2}}\right)ln\Phi_{2}+{\chi \Phi }_{1}\Phi_{2}$$

where *r*_1_ and *r*_2_ represent the number of monomer units in each polymer, and χ is the unitless FH interaction parameter that relates the strength of the API–polymer interaction. Equation (6) describes the mixing of two polymers, and in the case of API–polymer mixing, components 1 and 2 are drug and carrier, respectively, and *r*_1_ = 1. The first two terms of Equation (6) (right-hand side) relate to the combinatorial entropy of mixing, while the last term corresponds to the enthalpic contribution. Since mixing generally increases entropy, it is assumed that the dominance of the interaction parameter χ within the enthalpic term decides miscibility. The key threshold is χ = 0.5, with values of χ > 0.5 indicating likely phase separation between the two components, while χ < 0.5 predicts API–polymer miscibility. Negative values of χ further indicate that drug–polymer forces are greater than self-association forces (API–API, polymer–polymer). 

## 3. Molecular Modeling Approaches

Upon dispersion of the API within the polymer carrier, hydrogen-bonding, acid–base/ionic, dipole–dipole, and van der Waals non-bonding interactions discourage self-association, increase API–carrier interaction energy, and promote ASD physical stability over time [24,30,44]. Understanding miscibility between a given API and carrier requires the determination of the extent and types of interactions taking place between APIs and carriers in the ASD matrix. While this is a challenging task from a modeling perspective, as the carriers are large, highly flexible, multi-functional polymers, there are several molecular modeling techniques and software available for studying ASDs in detail (Table 2). The following sections describe quantum mechanics (QM) and molecular mechanics (MM)/molecular dynamics (MD) approaches for investigating ASD intermolecular interactions, solubility and FH miscibility parameters, formation, and stability.

### 3.1. QM Calculations for Elucidating Non-Bonding Interactions

QM methods such as density functional theory (DFT) provide insight into the non-bonding interactions taking place between API and polymer carriers through direct construction of the hypothesized molecular complexes and subsequent calculation of the associated interaction energy. Recent examples of ASD non-bonding interaction modeling highlight DFT as a relatively rapid (as compared to molecular dynamics; see Section 3.2.1) yet powerful computational methodology for predicting the interaction energy of model complexes and the identification of ionic and strong hydrogen-bonding pairs. As a computational modeling tool for studying solid-state pharmaceutical compounds, periodic DFT computations have seen increased applications in the literature [82]. Furthermore, combined DFT/MD methods may also be applied (e.g., Car–Parrinello MD) in which standard empirical force fields (see Section 3.2.1) are replaced with an ab initio description of the electronic structure [83,84,85,86]. Pure QM approaches commonly seen in ASD modeling produce a static snapshot and therefore require adequate, often manual, sampling of the conformational space as a prerequisite. Additionally, in order to facilitate DFT calculations of the large chemical systems, polymer molecules can be truncated to their monomeric or dimeric forms to model systems, which drastically reduces the time of the calculation. This reduction of calculation runtime as a result of the truncated model system allows for more systematic and detailed structural investigations but ultimately presents a tradeoff between a chemically realistic system and computational cost. Simulating a larger and more structurally realistic system does not necessarily result in an increase in accuracy but remains a desirable objective in ASD molecular modeling due to the ability to predict a higher number of properties and more diverse chemical behavior.

Miscibility and stability both depend on strong interactions between API and carrier, and understanding the critical interactions controlling ASD stability and solubility is a crucial step toward rational excipient screening and ASD formulation [87]. DFT applications in this context probe hydrogen-bonding and charge interactions. Maniruzzaman et al. investigated the interactions between cationic propranolol hydrochloride and diphenhydramine hydrochloride APIs with anionic Eudragit L100 and Eudragit L100-55 polymer carriers [46]. The polymer compounds were represented by their monomer forms, with multiple arrangements between monomer and polymer constructed, considering both the absence and presence of an explicit chloride anion within the complex. DFT computations found that the hydroxyl and amine groups of the API formed favorable hydrogen bonds with the ester and hydroxyl groups of the monomer units. Inclusion of the chloride ion in the complex was found to disrupt hydroxyl group interactions, but not amine group hydrogen-bonding, suggesting that the optimal interaction is between the amine groups of propranolol and diphenhydramine with the carbonyl groups of the L100 and L100-55 polymers. The proposed hydrogen-bonding motif was confirmed using nuclear magnetic resonance (NMR) and X-ray photoelectron spectroscopy experiments. Maniruzzaman et al. built upon the monomeric findings with a systematic study of dimeric API–carrier hydrogen-bonding interactions [47]. DFT computations showed 5 to 8 kcal/mol increase on average in binding energy between API and polymer for each additional hydrogen-bonding interaction, with the most stabilizing interaction being tertiary amine and carboxyl hydrogen-bonding (20 to 28 kcal/mol). In similar work, Nie et al. probed the molecular interactions between the antileprosy therapeutic clofazimine with hypromellose phthalate as carriers within an ASD system [48]. Partial charge analysis identified potential strong donors and acceptors, and a truncated model system with acetic acid as a structural proxy for the hypromellose phthalate carboxylic acid was used for DFT geometry and energy calculations. Based on these calculations and spectroscopic studies, formation of an ion pair complex was proposed as critical for API–carrier miscibility.

In an example of DFT calculations providing insight into ASD storage stability, Wang et al. studied the ability of polyvinylpyrrolidone (PVP) to inhibit crystallization of PVP–resveratrol and PVP–griseofulvin ASDs [88]. DFT calculations and Fourier-transform infrared (FTIR) spectroscopy investigated relative API–API and API–polymer interaction strengths, revealing greater stabilizing hydrogen-bonding interactions within the resveratrol complex than the griseofulvin–PVP system. The stronger interaction energy translated to higher stability, measured as a continued high dissolution rate after 90 days of storage of the PVP–resveratrol system compared to the lower dissolution rate of the griseofulvin–PVP ASD.

### 3.2. Molecular Mechanics and Molecular Dynamics Methods

With the advent of computing power over the last decade, MD simulations of amorphous API–API [89,90], API–micelle [91], polymer–polymer [92,93,94,95], polymer–membrane [96], and polymer–plasticizer [97] are prominent throughout the literature. The scope of the following sections focuses specifically on MD simulations of ASD systems composed of APIs interacting with polymer carriers. As applied to ASDs, MM-based docking and MD simulations predict a range of phenomena and properties. Recent literature examples are detailed pertaining to docking simulations probing API–carrier interactions, MD simulations to generate solubility and FH interaction parameters as predictors of API–carrier miscibility, and MD simulations probing ASD formation, polymer interactions with API crystals, and ASD hydration and dissolution mechanisms.

#### 3.2.1. Overview of Molecular Dynamics

Studying systems as large and complex as ASDs often necessitates the use of MD in order to approach a more physically realistic view of the phenomena under study. Application of MD requires that all components of the system are parametrized by a MM–based force field dictating the total energy as a sum of empirical potential energy functions (potentials) [98]. Common force fields applied to API–excipient ASD modeling (Table 3) are Condensed-phase Optimized Molecular Potentials for Atomistic Simulation Studies (COMPASS) [99,100], Polymer Consistent Force Field (PCFF) [101], CHARMM [102,103], Amber [104,105], and Optimized Potential for Liquid Simulations (OPLS) [106]. The force field describes energy as dependent on atomic position, and its subcomponent potentials can be divided into two types: (1) those for bonded atoms, describing bond lengths, angles, torsions, and (2) those for non-bonded atoms, accounting for Coulombic and van der Waals interactions. MD adds a temporal component to a molecular system by probing chemical phenomena over time. Chemical phenomena critical to predicting API–carrier miscibility and molecular mobility [107,108], such as hydrogen bond formation or motion of flexible chains, can emerge and recede over the course of the simulation and can be taken as average values over the simulation length (typically nanoseconds to microseconds) and even as averages from multiple runs originating from different initial states. Classical MD follows Newtonian laws of motion, tracking atomic position, velocity, acceleration, force, and energy across all atoms for each time step of the simulation. The total energy at a given time is then a function of atomic positions, where the positions are updated for each time step of the simulation in an iterative fashion based on the forces (and therefore acceleration) encountered by the atoms.

#### 3.2.2. Docking Studies of API with Polymer Carrier

Docking provides an algorithmic method for rapidly sampling and scoring complexes between API and polymer carriers and, within this context, is often used to generate favorable preliminary binding conformations, which are then subjected to more in-depth analysis and energy refinement through MD simulations. Barmpalexis et al. investigated ternary ASD systems of ibuprofen and carbamazepine APIs, Soluplus (SOL) polymer, and polyethylene glycol (PEG) plasticizer [50]. As with QM approaches, monomeric or dimeric model systems of the polymer are often employed to probe specific interactions and reduce computational cost. Fule et al. undertook docking simulations to investigate intermolecular interactions between the antimalarial lumefantrin with monomers of SOL, Kollidon VA-64, and vinylpyrrolidone-vinyl acetate polymers [78]. Strong hydrogen-bonding was identified between SOL and vinylpyrrolidone-vinyl acetate monomers and lumefantrin hydroxyl, chlorine, and amine groups. Docking can be used not only to identify potential non-bonding interactions but also to rapidly screen the stability of potential API tautomeric forms prior to MD simulations. Gangurde et al. applied docking to assess the binding energy of diketo and keto-enol forms of curcumin with Eudragit EPO monomers and dimers [79], finding that the diketo tautomer was overall favored, with binding controlled primarily by van der Waals interactions. These initial docking studies were followed by MD simulations to refine the docking energies and provide more detailed structural insight into curcumin–Eudragit EPO interactions. In a comprehensive docking study, Macháčková et al. detail systematic docking simulations with full-size polymers [60]. In this study, the anchoring ability of cyclosporin A was investigated with short (~7 nm), medium (13–14 nm), and long (~20 nm) chain polymers of l/d–polylactide, chitosan, polyglycolic acid, PEG, and cellulose. For each type of polymer modeled at a given length, one million complexes were generated between stationary cyclosporin A and flexible polymer, and the resulting interaction energies predicted chitosan and cellulose as most miscible with cyclosporin A.

#### 3.2.3. Theory of MD-Derived Solubility and FH Interaction Parameters

In the context of MD atomistic simulations, cohesive energy density and solubility parameters are derived by comparing the total energy of the system in a vacuum (infinitely separated) to the interacting, modeled amorphous state (bulk). This difference in energy relates to the energy of vaporization, and the MD-derived Hildebrand solubility parameter is taken as Equation (7) [109]:

(7) $$\delta^{2}=\frac{E_{\mathrm{vacuum}}-E_{\mathrm{bulk}}}{V}$$

where *E*_vacuum_ is the potential energy of a single system component (API or polymer) in vacuum, *E*_bulk_ is the potential energy of the same component in the simulated system, and *V* is the volume of the periodic cell. Since MD force field potentials explicitly account for Coulombic and van der Waals forces, the predicted solubility parameter can be further decomposed into its non-bonding electrostatic and dispersion terms, yielding the Hansen solubility parameter. From MD-derived electrostatic (accounting for both polar and hydrogen-bonding interactions) and dispersion contributions to the total potential energy, the Hansen solubility parameter is given by Equation (8):

(8) $$\delta^{2}=\frac{\Delta E_{e}+\Delta E_{d}}{V}$$

where *E*_e_ and *E*_d_ are the electrostatic and dispersion contributions to the potential energy, respectively, between infinitely separated molecular components and the simulated interacting molecular assembly for all constituents of the system (∆*E* = *E*_vacuum_ − *E*_bulk_) [68,74]. MD simulations can also be used to predict FH interaction parameters. The computed cohesive energy densities (*CED* = ∆*E/V*) are related to the energy of mixing, ∆*E*_mix_, and finally to the FH interaction parameter, χ, via Equation (9):

(9) $$\Delta E_{\mathrm{mix}}=\Phi_{1}CED_{1}+\Phi_{2}CED_{2}-CED_{\mathrm{mix}}$$

and Equation (10):

(10) $$\chi =\frac{\Delta E_{\mathrm{mix}}}{RT}$$

where the Φ terms represent the volume fractions of the individual components within the mixed system. It should be noted that the Flory–Huggins model does not account for entropic effects related to the structural properties of the polymer. Entropic energetic penalties such as restricted polymer movement within the ASD matrix from API–carrier intermolecular hydrogen-bonding are not explicitly accounted for. Accurate calculation of the entropic contribution is considered a significant cause of the observed discrepancies between predicted and experimental miscibility evaluations (see end of Section 3.2.4) [110].

#### 3.2.4. Applications of MD-Derived Solubility and FH Interaction Parameters

MD methodology provides an attractive and versatile approach for calculating solubility and FH interaction parameters (Table 3). Gupta et al. applied MD simulations to compute solubility parameters and compared the MD-derived values with solubility parameters calculated using the Hoftyzer–Van Krevelen and Hoy group contribution methods [57]. Calculated solubility parameters predicted that indomethacin API was miscible with polyethylene oxide (PEO), borderline miscible with sucrose, and immiscible with glucose. These predictions agreed with differential scanning calorimetry (DSC) experiments, which showed indomethacin miscibility with PEO and general immiscibility with sucrose and glucose carriers. Yani et al. investigated ibuprofen, fenofibrate, and alprazolam API miscibility with poly(vinylpyrrolidone-co-vinyl acetate) 64 (PVP–VA 64), hydroxypropyl methylcellulose (HPMC), and Eudragit EPO polymer carriers using Hildebrand and Hansen solubility parameters [63]. MD simulations were used to derive Hansen solubility parameters and to calculate the average hydrogen bond lifetime and total hydrogen bond interaction energy per monomer between API and polymer functional groups. Based on the predicted solubility, hot melt extrusion was used to produce ASDs for ibuprofen/PVP–VA 64, ibuprofen/Eudragit EPO, and fenofibrate/PVP-VA 64. Weaker predicted relative intermolecular interactions in the fenofibrate/PVP–VA 64 ASD coincided with its observed recrystallization. Barmpalexis et al. took the most stable poses from docking simulations between ibuprofen and carbamazepine binding with SOL/PEG polymer/plasticizer blends (Section 3.2.2) and evaluated miscibility using Hoftyzer–Van Krevelen and Hildebrand solubility parameters [50]. Both ternary systems were shown as miscible from calculated solubility parameters and DSC experiments. FTIR spectroscopy was applied to probe the intermolecular interaction within the ASD and confirmed strong hydrogen-bonding observed from docking and MD between the carbamazepine primary amine group and the SOL carbonyl and amide groups. MD studies by Iesavand et al. calculated the enthalpy of mixing (∆*H*_mix_) and Hildebrand solubility parameters of 6-Mercaptopurine API with polylactic acid (PLA) and PEG-modified PLA [58].

The FH interaction parameter has also seen recent application for probing API–carrier miscibility in combination with MD simulation. Macháčková et al. used insights from docking (Section 3.2.2) and MD–derived FH interaction parameters to identify chitosan and cellulose as the most miscible polymers with cyclosporin A [60]. Additionally, MD probed the polymer flexibility as measured by radius of gyration and elucidated all hydrogen-bonding interactions within the API–polymer complexes, revealing that miscibility primarily correlated with polymer chain length, cyclosporin A–polymer interaction energy, and the number of hydrogen bonds between cyclosporin A and polymer. Xiang et al. computed both solubility and FH interaction parameters between amorphous systems of indomethacin and PVP, varying API, polymer, and water composition in each MD simulation [68]. While differences in the solubility parameters of indomethacin and PVP predicted close to borderline miscibility (*δ*_IMC-PVP_ = 6.5 MPa^1/2^), FH interaction parameters predicted complete miscibility (χ_IMC-PVP_ = − 0.61). In later work, Xiang et al. similarly calculated FH parameters for ASDs of felodipine API and HPMC polymer for several compositions of API, polymer, and water, with both predicted solubility and FH parameters showing complete miscibility across all modeled compositions [69]. Erlebach et al. investigated mixtures of indomethacin with PEG and PLA polymers, studying ASD formation from simulated annealing, API–polymer miscibility from MD–predicted FH interaction parameters, and encapsulation efficiency of the polymer carrier [56]. Eslami et al. calculated both solubility and FH interaction parameters for tacrine interacting with chitosan and polybutylcyanoacrylate (PBCA) polymers [74]. Tacrine showed higher miscibility with PBCA with both approaches, and the MD simulations further revealed that increases in polymer chain length result in higher MD–derived interaction energies. In a recent study, Kapourani et al. disclosed a computational and experimental evaluation of ASDs formed between simvastatin API and PVP [52]. MD-computed Hansen solubility and FH interaction parameters predicted miscibility, which was verified using DSC experiments.

While MD–derived miscibility assessments provide utility and often complement experimental results, the accuracy of these methods remains largely untested as direct comparisons between MD–predicted solubility and FH interaction parameters and experimental values are uncommon. Rather, comparisons to experimental results typically rely on more qualitative observations. Examples include FTIR confirmation of strong intermolecular interactions and single observed *T*_g_ values for ASDs predicted as miscible, or the onset of rapid ASD recrystallization for a system predicted as immiscible (or even complete lack of ASD yield upon synthesis). Within a small scale of studied APIs and carriers, predicted and observed trends tend to agree (Table 3). With respect to quantitative experimental comparisons, Turpin et al. disclosed a systematic screen of API–carrier ASDs, allowing direct evaluation of predicted and experimental miscibility parameters [110]. High-throughput assays measured the miscibility limits for combinations of nine APIs and six polymer carriers (poly (glycerol–adipate) (PGA), three PGA derivatives, PVP, and PVP–VA 64). Both MD-predicted solubility and FH interaction parameters showed poor correlation with experimental miscibility. For FH interaction parameters predicted with PGA carrier, six of the nine API/PGA mixtures were predicted as having complete miscibility (χ < 0), in contrast to experimental results.

### 3.3. Mechanistic Insights from Molecular Modeling

**Figure 2:**
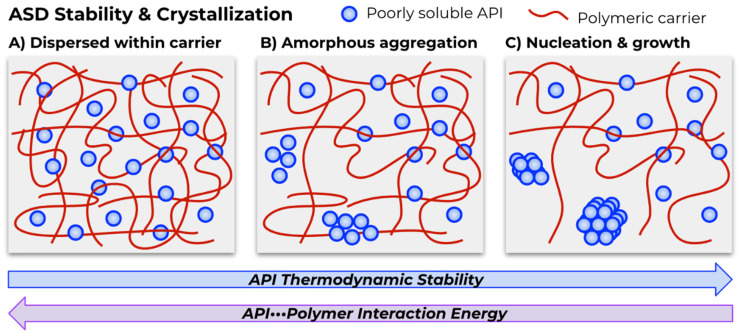
(**A**) Thermodynamic stabilization from API–polymer non-bonding interactions promotes API dispersion throughout the polymer carrier matrix. (**B**) Molecular mobility can lead to increased API–API interaction and aggregation. (**c**) Small sites of local aggregation and nucleation can grow to larger API crystals incurring reduced solubility.

Knowledge pertaining to the mechanisms and rates of API dissolution [111], ASD formation, and ultimately ASD dissolution and drug release [112] from the carrier matrix is of great value to guide and streamline candidate excipients for a given poorly water-soluble therapeutic, resulting in lower development times and faster progression to clinical trials. Dissolution and drug release are highly mechanistically complex processes, with three underpinning hypotheses [19,20]: (1) rapid dissolution forming a supersaturated solution that promotes the generation of amorphous or crystalline API nanoclusters within the polymer carrier, (2) gradual release of amorphous API–polymer complexes from the greater bulk ASD complex, and (3) gradual release of API–polymer, but plasticization by water causes crystalline API–polymer clusters to form at the ASD–solvent interface. Which cases apply to a given ASD depends on the properties of the API, carrier, and the ASD formulation. In tandem with more time-consuming and resource-intense experimental mechanistic studies, recent literature examples show that computational methods can provide detailed insight into mechanistic aspects of ASD formation and dissolution [27]. Chan et al. applied MD simulations to the investigation of formation and dissolution of ASDs composed of ibuprofen and PEG, PVP, and poloxamer 188 (P188) [66]. ASD formation was modeled using the simulated annealing method followed by water immersion to investigate dissolution behavior, with small solid dispersion particles displaying rapid ASD dissolution. For larger particles, the dissolution behavior was dependent on the polymer. Higher API mobility within PEG and PVP polymers tended towards API aggregation, a precursor for API recrystallization (Figure 2), while P188 slowed API aggregation. A combined experimental and molecular modeling approach by Han et al. investigated a ternary ASD system containing glipizide API, PEG carrier, and sodium dodecyl sulfate/Tween 80 (Tween 80 is derived from polyethoxylated sorbitan and oleic acid) surfactants [67]. MD simulations studied ASD formation (simulated annealing), molecular mobility, and ASD dissolution.

Maintaining the supersaturated high energy state of ASDs increases solubility for poorly water-soluble APIs, but ASDs are not an equilibrium system, and there is a constant pull towards the thermodynamic stability of the API crystalline phase (Figure 2). The kinetic interplay between API dissolution and crystallization requires careful tuning of the properties of polymer carriers that promote extremely rapid dissolution, as fast API dissolution can lead to crystallization due to rapid supersaturation [113,114].

ASD formulation design decisions seek API–excipient mixtures that will inhibit or prolong crystallization, providing optimal long-term storage and physical stability, but modeling and predicting crystallization is highly challenging. The methodologies outlined in Section 3.1 and Section 3.2, related to quantifying the relative strength of API–polymer interaction and miscibility prediction of solubility and interaction parameters, are thermodynamic evaluations, giving no kinetic information on crystallization [115]. Furthermore, MD simulations that truly capture phase separation events when modeling API–excipient mixtures would need to run for exceedingly long simulation time scales in order to increase the likelihood of observing such phenomena. A recent example by Brunsteiner et al. discloses MD simulations of eight different API–polymer combinations of phenacetin and flufenamic acid APIs with Eudragit, polystyrene sulfonic acid, poly acrylic acid, and PVP polymers [72]. Thermodynamic mixing energy, kinetic diffusivity, and mobility were both investigated in detail, and this work highlights the importance of obtaining thermodynamic and kinetic information for confidence in ASD stability over time. As the 20 microsecond simulations of ~20,000 atoms took months to complete on standard computing machines, adapting and scaling such an approach for industrial and pharmaceutical applications such as API–excipient screening and long-term physical stability assessments currently remains limited by computational power. API crystalline lattices may also be constructed and modeled directly as an approach to furnish insight into ASD crystallization. Gao et al. studied interactions of diblock copolymer poly(ethylene glycol)-block-poly(lactic acid) (PEG-*b*-PLA) at the interface of tolazamide crystal slabs in aqueous conditions [76]. MD simulations found that PEG-*b*-PLA interacted most rapidly and strongly with the (001) face, followed by the (010) face, with minimal interactions at the interface of the (100) crystal face.

Of significant importance to ASD development and formulation are the effects of water on ASD stability and dissolution rate, with direct implications for both understanding moisture uptake during storage and in vivo solubility and absorption. Jha et al. used in-depth MD simulations to probe the intermolecular interactions and dissolution behavior between poorly soluble phenytoin API and cellulose-type polymer excipients [59]. ASD dissolution behavior was investigated by varying water weight percentages and tracking the rate of phenytoin diffusion, where diffusion constants were determined to have an exponential relationship with increases in water weight. Razmimanesh et al. investigated the effects of hydration on API loading efficiency from MD simulations of gemcitabine API and chitosan polymer carrier with varying concentrations of API [61]. Both the maximum drug loading efficiency and the strongest intermolecular interactions between gemcitabine and chitosan were predicted for the intermediate API concentration (40%) in simulated aqueous conditions. In addition to FH interaction parameters (Section 3.2.4), MD simulations by Xiang et al. studied the effects of hydration on hydroxypropyl methylcellulose polymer physical properties, finding that increasing water fractions lead to decreased *T*_g_ and increased structural relaxation and molecular mobility [69]. Recent work has also pointed to the role of solvation free energy in inhibiting API crystallization. Mosquera-Giraldo et al. investigated ASDs formed from telaprevir API and a range of synthesized cellulose polymer derivatives [54]. QM free energies of hydration were computed for cellulose monomers from application of implicit solvation using the polarized continuum model for water. Monomers with more favorable free energies of hydration corresponded to cellulose polymers yielding more stable ASDs with telaprevir, highlighting that polymer ionization is important not only for intermolecular API–polymer interactions but also for solubilizing the carrier itself.

## 4. Machine Learning Approaches

### 4.1. Overview of Machine Learning

Many of the complexities seen experimentally and via molecular modeling when delineating ASD formation, stability, and dissolution mechanisms can potentially be overcome using statistical learning models. Machine learning (ML), a subset of artificial intelligence (AI), has been at the forefront of major speed and efficiency improvements among in silico drug development approaches over the last decade. By enabling the extraction of complex and often non-linear relationships between the input features and the target feature, ML has been increasingly popular in diverse areas of healthcare and pharmaceutical [116,117,118] and chemical research applications [119,120]. With increasing amounts of data available experimentally, data-driven supervised ML and AI algorithms have shown progress in developing effective drug formulation models [121,122,123]. Even with sparse data, there has been a rise in ML algorithms such as transfer learning, one-shot, zero-shot learning, and Bayesian-based optimization approaches to improve the model performance given small amounts of data [124,125,126].

A subfield of ML is deep learning (DL) [127], exemplified by artificial neural networks (ANNs). ANNs mimic the neural connectivity of the brain, with all nodes (neurons) connected to every other node in the network either directly or indirectly through the layers. ANNs take in information from the input layer, which is then processed through one or more connected hidden layers and finally sent as responses to the output layer. ANNs are especially advantageous for deciphering non-linear and complex unknown relationships between input and output variables. Amongst the many available ML methodologies, ANNs have shown increasing and diverse applications to drug development and process optimization [128,129] and appear in the majority of recent disclosed ML models for predicting and optimizing ASD composition, stability, and dissolution rate (Table 4).

Often, many ML algorithms are applied to the same problem, allowing prediction accuracy of the same target trained on identical data to be compared across multiple algorithms, as seen by Han et al. in Table 4 [130]. In addition to ANNs, other ML methods surveyed in this review include genetic algorithms (GA) [131], multiple linear regression (MLR), logistic regression (LR) [132], decision tree (DT) [133], random forest (RF) [134], k-nearest neighbors (kNN) [135], Naïve Bayes (NB) [136], and light gradient boosting machine (LGBM) [137].

### 4.2. ML Applications to General ASD Systems

There are relatively few examples of statistical learning models predicting properties and phenomena of ASDs formed between small-molecule APIs and polymer carriers, likely due to limited experimental data for model training. ML models tend to focus on predicting target features of amorphous small-molecule compounds. Nevertheless, ML models developed for predicting amorphous API properties provide important understanding and analysis towards rational API–polymer ASD development and formulation [142]. As an example, Nurzyńska et al. developed MLR models for predicting the long-term physical stability of the amorphous forms of 25 poorly water-soluble compounds (no carrier, only the amorphous form of the drug compound) using physicochemical properties directly from two-dimensional structure as input features in addition to measured thermodynamic and kinetic solid properties such as melting point, glass transition temperature, enthalpy of fusion, relaxation time, and configurational free energy [143]. Features that correlate with predicting the amorphous behavior of pure APIs may also show importance in prediction models of API–carrier ASD systems. In another model solely focused on small molecule solubility properties, Przybyłek et al. constructed a model to predict Hansen solubility parameters from a dataset of 130 compounds for which measured solvent solubility parameter data were available [144]. A large collection of connectivity features, indices, and physicochemical properties were generated directly from SMILES (simplified molecular-input line-entry system) data and used as input features to train multivariate adaptive regression splines for solvent solubility parameter prediction. The model was further extended to solvent–polymer miscibility predictions via a binary classification model and predicting solvent-dependent drug-like solid dissolution.

### 4.3. ML Models of ASD Properties and Phenomena

In addition to thermodynamics and molecular modeling approaches, ML has also been applied to evaluating the likelihood of ASD formation between a given API and polymer carrier. Moore et al. developed a LR model to predict the potential of ASD formation using data from twelve small-molecule therapeutics co-solidified with PVP/vinyl acetate copolymer as a stabilizing agent [139]. The target feature, ASD formation potential, was determined by experimental characterization of miscibility and stability, with mixtures that exhibited rapid crystallization or phase separation deemed immiscible. Topological and molecular indices were generated from atom connectivity and three-dimensional structures of the small-molecule compounds. The most significant descriptors identified for predicting dispersion potential within the dataset were the atomic mass-weighted third-order R autocorrelation index and the topological distance (i.e., number of path-connected bonds) between oxygen and chlorine atoms.

The optimal ratio for all components within the ASD is critical for rational formulation design and relates to ASD physical properties, stability, and drug release behavior. Furthermore, ASD forms and subsequent properties are sensitive to small changes in starting material ratios during synthesis and formulation. Predicting such properties and behavior is challenging for binary mixtures and becomes increasingly challenging for systems with even more components. Barmpalexis et al. approached this optimization problem using the statistical learning methodologies of ANNs and GAs. ASDs of nimodipine API with PEG polymer carrier were characterized experimentally, and the ANN and GA models trained to optimize physical formulation and drug release properties of effervescent controlled release floating tablets [138]. The ANN input layer consisted of PEG, PVP, HPMC, effervescent, and nimodipine proportions within the formulation, and an output layer with responses for percent drug released at 60 min, time to reach 90% dissolved, float strength, and float duration. Inclusion of trigonometric and exponential/logarithmic functions when deriving the GA equation accounted for complex non-linearity. Input feature analysis indicated the proportion of effervescent agents as most important, followed by percent HMPC, while PEG and PVP proportions as features showed minor importance in the model. Applying a similar approach, Medarević et al. constructed an ANN model to predict the dissolution rate of carbamazepine API within ternary ASDs of SOL and P188 carriers [141]. Carbamazepine release rate was found to correspond most strongly with ASD compositions lowest in carbamazepine and highest in P188. ASD dissolution rate was also modeled by Barmpalexis et al. by constructing an ANN to predict the rate of dissolution (measured as percent API dissolved after thirty minutes) of ASDs formed from mixtures of tibolone and different molecular weights of PEG polymers [140]. Dissolution rate was determined to primarily correlate with percent tibolone in the mixture, molecular weight of the PEG polymer, and the mixing temperature.

The long-term physical stability of ASD formulations remains a significant challenge, and one that is often not sufficiently addressed by solubility and interaction parameters alone [110]. Given specific data related to ASD stability over time, machine learning models can be trained to directly predict ASD stability and dissolution. In a recent study, Han et al. combined molecular modeling techniques and eight different machine learning algorithms towards the development of models for ASD physical stability prediction [130]. The collected ASD formulation data encompassed formulation, process parameters, stability measurement conditions, and experimental stability measurements (“stable” vs. “unstable” labels after both three and six months). The random forest (RF) algorithm gave the highest accuracy models, and RF feature importance analysis determined drug loading ratio, relative humidity, temperature (storage and preparation), and molecular weight (polymer carrier and API) as key features for the prediction of three- and six-month physical ASD stability.

## 5. Conclusions and Outlook

Formulating poorly water-soluble drugs as ASDs with polymeric carriers is an effective method for increasing solubility yet is often hindered by complex characterization experiments and lengthy development cycles. It is likely that the representation of approved ASDs coming to market will remain relatively low until significant advances are achieved in predicting compatible carriers and excipients for novel therapeutic compounds with poor aqueous solubility. Much attention has been given to the use of solubility and FH interaction parameters for estimating API–polymer thermodynamic miscibility. Molecular modeling via MM, QM, and MD approaches for studying API–polymer miscibility and intermolecular non-bonding interactions, in addition to investigating mechanisms of ASD formation, physical stability, dissolution, and drug release, have increased alongside advances in computational power. These studies have provided a breadth of information but are often limited to a small number of APIs and carriers. More large-scale studies with quantitative comparisons between predicted and experimental results will assist in theoretical progress, in which a remaining major challenge is the accurate calculation of the entropic contribution to miscibility. Furthermore, combined DFT–MD methods providing an electronic description of molecular systems remain highly underexplored in studies of ASD API–polymer interactions and may also yield improved model predictions. Finally, even today’s computational power remains a limiting factor in the simulation of important ASD kinetic behavior such as molecular mobility and mechanisms of phase separation and crystallization over desired storage and physiologically relevant timescales, all of which dictate long-term ASD physical stability. These limitations must be overcome prior to ASD computational modeling tools arriving as more mainstay applications in process chemistry and pharmaceutical settings.

ML methods have the potential to provide the next transformative leap forward toward rapid polymer screening and formulation design, with recent examples already showcasing the ability of ML to predict API–polymer miscibility, optimize ASD formulation for desired physical properties, and predict dissolution rates. Of special note for future applications and expanded research is close integration of molecular modeling and machine learning pipelines [145]. Prediction accuracy and model robustness will only improve over time as more and more data become available, where data encompass both experimental targets and more representative chemical and physical input descriptors of ASD molecular systems. Many experimental and molecular modeling studies test less than ten polymers for a given API, with a fraction of the combinations yielding promising results, yet an advantage of ML is that negative outcomes remain valuable as the training set should avoid skew towards only positive experimental results. In this sense, almost all characterization data and excipient screening results are pertinent to ML model development, and the limiting factors become data organization, labeling, and public accessibility.

The overarching driving force of ASD modeling is ultimately to bring more poorly soluble therapeutics to market in order to rapidly combat much needed disease areas. Conventional approaches to excipient screening and ASD development have relied on trial and error experiments, both to match a given API with a compatible polymer and to further optimize the formulation properties once a suitable match is located. There remains only around thirty commercial ASD formulation products available on the market (Table 1). Experimentally, it is intractable to survey even half of all available polymers and excipients, with potential synthesized novel carriers expanding the chemical space further. This bottleneck necessitates that computational approaches continue to play a vital role. Advances in predictive ASD models will facilitate more widespread rational excipient screening, promote de novo excipient construction, and accelerate ASD development, thus expediting the progression from early-stage assessment and characterization to stable formulations with optimal physical properties primed for preclinical and clinical testing.

## Figures and Tables

**Table 1:** Summary of commercially available ASD formulations.

Product	API	Carrier	Dosage Form
Afeditab	Nifedipine	Poloxamer/PVP	Tablet
Afinitor	Everolimus	HPMC	Tablet
Certican	Everolimus	HPMC	Tablet
Cesamet	Nabilone	PVP	Tablet
Crestor	Rosuvastatin	HPMC	Tablet
Cymbalta	Duloxetine	HPMC-AS	Capsule
Fenoglide	Fenofibrate	PEG	Tablet
Florfenicol	Florfenicol	Cellulose acetate phthalate	Powder
Gris-PEG	Griseofulvin	PEG	Tablet
Incivek	Teleprevir	HPMC-AS	Tablet
Incivo	Etravirine	HPMC	Tablet
Intelence	Etravirin	HPMC	Tablet
Isoptin	Verapamil	HPC/HPMC	Tablet
Kaletra	Lopinavir	PVP	Capsule
Kalydeco	Ivacaftor	HPMC-AS	Tablet
Nimotop	Nimodipine	PEG	Capsule
Nivadil	Nivaldipine	HPMC	Tablet
Novir	Ritonavir	PVP	Tablet
Onmel	Itraconazole	HPMC	Tablet
Prograf	Tacrolimus	HPMC	Capsule
Rezulin	Troglitazone	HPMC	Tablet
Shuilinjia	Silibinin	Lecithin	Capsule
Sporanox	Itraconazole	HPMC	Capsule
Stivarga	Regorafenib	HPMC	Tablet
Votubia	Everolimus	HPMC	Tablet
Zelboraf	Vemurafenib	Hypromellose acetate succinate	Tablet
Zortess	Everolimus	HPMC	Tablet

PVP: polyvinylpyrrolidone; HPMC: hydroxypropyl methylcellulose; HMPC-AS: hydroxypropyl methylcellulose acetate succinate; PEG: polyethylene glycol.

**Table 2:** Molecular modeling software applied in this review to studying ASD systems.

Software	Applicability	License	Reference(s)
Gaussian [45]	MM and QM computations	Commercial	[46,47,48]
AutoDock Vina [49]	MM conformational sampling docking	Apache License	[50]
XenoView [51]	MM and MD simulations	Non-commercial	[50,52]
HyperChem [53]	MM, QM, and MD simulations	Commercial	[54]
Materials Studio (BIOVIA) [55]	MM, QM, and MD	Commercial	[56,57,58,59,60,61,62,63]
Amber [64,65]	MM and MD simulations	Proprietary ^1^	[66,67,68,69]
GROMACS [70,71]	MM and MD simulations	LGPL	[54,59,72]
LAMMPS [73]	MM and MD simulations	GPL	[74]
NAMD [75]	MM and MD simulations	Proprietary, free for noncommercial use	[50,76]
Maestro (Schrödinger) [77]	Molecular modeling	Commercial	[78,79]
Desmond (Schrödinger Materials Science Suite) [80]	MM and MD simulations	Commercial	[81]

^1^ GPL for AmberTools. MM: molecular mechanics; QM: quantum mechanics; MD: molecular dynamics; GPL: General Public License; LGPL: Lesser General Public License.

**Table 3:** Recent studies of ASD API–polymer miscibility from MD-derived solubility and interaction parameters.

APIs	Polymer Carriers	Miscibility Parameter(s) Investigated	Force Field	Brief Simulation Overview ^1^	Experimental Miscibility Comparison	Reference
Indomethacin	PEO, glucose, sucrose	*δ* ^2^	COMPASS	2 ns NVT ^3^/NPT ^4^ equilibration, 200–500 ps NVT production (298 K, 1 fs/step)	PEO (miscible), glucose (immiscible), sucrose (borderline) predictions in agreement with thermal analysis experiments.	[57]
Artemisinin	PVP/PEG	δ	COMPASS	500 ps NPT equilibration, 200 ps production (298 K, 1 fs/step)	Predicted PVP and PEG miscibility in agreement with observed drug dispersion from thermal analysis.	[62]
Gemcitabine	Chitosan	δ	COMPASS	200 ps NPT equilibration, 800 ps production (298 K, 1 fs/step)	N/A	[61]
Telaprevir	Cellulose derivatives	δ	CHARMM	50 ps NVE (0.5 fs/step), 5 ns NVT/NPT (310 K, 1 fs/step) equilibration, 40 ns NPT production (310 K, 1 fs/step)	N/A	[54]
Clonazepam, ibuprofen, fenofibrate, alprazolam	PVP–VA 64, HPMC, and Eudragit EPO	δ	COMPASS	2 ns NPT equilibration, 500 ps NVT production (298 K, 1 fs/step)	Predicted fenofibrate/PVP-VA 64 weaker intermolecular interactions in agreement with observed recrystallization during stability experiments.	[63]
Ibuprofen, carbamazepine	SOL/PEG	δ	CHARMM	2 ns NPT relaxation (393 K cooled to 298 K for ibuprofen, 474 K cooled to 298 K for carbamazepine, 10 K/step), 100 ps NPT equilibration/300 ps production at each temperature.	Both ibuprofen and carbamazepine predicted as miscible with SOL/PEG in agreement with observed single *T*_g_ values from DSC experiments.	[50]
6-Mercaptopurine	PLA, PEG-modified PLA	δ	PCFF	2 ns NPT dynamics (298 K, 1 fs/step)	N/A	[58]
Olmesartan medoxomil	PVP–VA 64, SOL	δ	OPLS	5 ns NPT dynamics (300 K, 1 fs/step)	Predicted high miscibility with PVP–VA 64 carrier reflected in crystallography and thermal analysis experiments.	[81]
Cyclosporin A	l/d–polylactide, chitosan, polyglycolic acid, PEG, cellulose	*χ* ^5^	PCFF	1.5 ns NPT dynamics (298 K)	N/A	[60]
Indomethacin	PEG, PLA	χ	COMPASS	5 ps NVT equilibration at each temperature step (298 K heated to 500 K in three steps, then cooled back to 298 K in three steps). 30 ns equilibration at the last step. 1 ns NPT production (298 K, 1 fs/step)	Predicted significant miscibility (negative interaction parameters) for indomethacin with both PEG and PLA as carriers in agreement with encapsulation efficiency experiments.	[56]
Felodipine	HPMC	δ, χ	Amber/GLYCAM	10 ns equilibration (500–700 K), then cooled to 200 K (0.03 K/ps). 30–100 ns production (298 K, 1 fs/step).	Predicted miscibility from solubility and interaction parameters at all HPMC concentrations in agreement with observed single *T*_g_ values from DSC.	[69]
Indomethacin	PVP	δ, χ	Amber	10 ns equilibration (600 K), then cooled to 200 K (0.03 K/ps). 100 ns production runs (298 K, 1 fs/step).	N/A	[68]
Tacrine	Chitosan, PBCA	δ, χ	PCFF	100 ps equilibration (300 K), 5 ns NPT (298 K, 1 fs/step)	N/A	[74]
Simvastatin	PVP	δ, χ	PCFF	5 ns NPT relaxation (600 K cooled to 200 K, 10 K/step, 1 fs/step). 400 ps NPT runs at each temperature. (1 fs/step)	Predicted miscibility from MD–based interaction parameter calculation in close agreement with measured value derived from melting point depression experiments.	[52]
Aspirin, caffeine, carbamazepine, finasteride, flufenamic acid, flutamide, mefenamic acid, salicylamide, theophylline	PVP–VA 64, poly (glycerol adipate) and derivatives	δ, χ	CHARMM	Iterate cell volume prior to arriving at target density. At each step of the cycle, minimization, then 200 ps NVT dynamics (700 K, 1 fs/step). Cell then underwent annealing from 750 K to 300 K (0.1 K/ps). Minimization then 10 ns NPT dynamics (300 K).	Predicted solubility and interaction parameters showed no correlation to measured miscibility limits. Opposite to experimental values, MD-derived FH interaction parameters for six of nine API–PGA polymers predicted complete miscibility.	[110]

^1^ See reference for more simulation details, including specific software settings, force field parameters, API–polymer cell construction, energy minimization algorithms, thermostats/barostats algorithms, periodic boundary conditions, integrators, dispersion cutoff distances, and long-range electrostatic methods. ^2^ Solubility parameter (δ), ^3^ Canonical ensemble conserving substance (*N*), volume (*V*), and temperature (*T*). ^4^ Isothermal–isobaric ensemble conserving substance (*N*), pressure (*P*), and temperature (*T*). ^5^ Flory–Huggins interaction parameter (χ). N/A: Not Applicable; PEO: polyethylene oxide; PVP-VA 64: poly(vinylpyrrolidone-co-vinyl acetate) 64; SOL: Soluplus; PBCA: polybutylcyanoacrylate; Differential scanning calorimetry: DSC.

**Table 4:** Recent ML prediction models for API–carrier ASD properties, stability, and formulation.

Year	Target Features	Input Features	Algorithms	Dataset	Model Evaluations	Reference
2011	API percent release after 60 min, time for 90% dissolved, tablet floating strength and time	Ratios of API/polymers/effervescent agents	ANN, GA	25 mixture proportions	Root mean squared error of prediction across each parameter ranges from 0.0184 to 0.0782 as evaluated on external validation set.	[138]
2011	Solid dispersion potential, *P*(Y) (miscibility)	Molecular and topological indices from atom connectivity and three-dimensional coordinates	LR	12 compounds co-solidified with polymer carrier	Best univariate regression model: logit *P*(Y) = −1.927 + 0.208T(O···Cl) giving deviance of 6.513, likelihood ratio of 10.86, and leave-one-out cross-validation error of 0.3841.	[139]
2013	Percent API dissolved after 30 min	Polymer molecular weight, temperature of melt mixing, total mixing time, percent API in the ASD	ANN	36 combinations of input features	*R*^2^ = 0.9896, *p*-value < 0.001, lack of fit *p*-value = 0.456, coefficient of variation = 3.53	[140]
2015	API percent release after 10 and 20 min	Ratios within ternary system of API and two polymers	ANN	25 mixture proportions	*R*^2^ = 0.978 (observed versus predicted)	[141]
2019	ASD physical stability after 3 and 6 months	Molecular weight, melting point, XlogP3, hydrogen bond donors and acceptors, rotatable bonds, polar surface area, heavy atoms, complexity, intrinsic solubility	ANN, DNN SVM, RF, DT, LGBM, kNN, NB	50 compounds with 646 ASD physical stability data	Best model (RF) gave test set prediction accuracy of 82.5% (3 and 6 months). NB was the least accurate model (46.67%), with all other models ranging from 70.83% to 80.83% for test set accuracy (3 and 6 months)	[130]

ANN: artificial neural network; GA: genetic algorithm; LR: logistic regression; DNN: deep neural network; SVM: support vector machine; RF: random forest; DT: decision tree; LGBM: light gradient boosting machine; kNN: k-nearest neighbors; NB: Naïve Bayes.

## References

[1] Amidon G.L., Lennernas H., Shah V.P., Crison J.R. (1995). A theoretical basis for a biopharmaceutic drug classification: The correlation of in vitro drug product dissolution and in vivo bioavailability. Pharm. Res..

[2] Butler J.M., Dressman J.B. (2010). The developability classification system: Application of biopharmaceutics concepts to formulation development. J. Pharm. Sci..

[3] Savjani K.T., Gajjar A.K., Savjani J.K. (2012). Drug solubility: Importance and enhancement techniques. ISRN Pharm..

[4] Meanwell N.A. (2011). Improving drug candidates by design: A focus on physicochemical properties as a means of improving compound disposition and safety. Chem. Res. Toxicol..

[5] Walker M.A. (2014). Novel tactics for designing water-soluble molecules in drug discovery. Expert Opin. Drug Discov..

[6] Arnott J.A., Planey S.L. (2012). The influence of lipophilicity in drug discovery and design. Expert Opin. Drug Discov..

[7] Manallack D.T., Prankerd R.J., Yuriev E., Oprea T.I., Chalmers D.K. (2013). The significance of acid/base properties in drug discovery. Chem. Soc. Rev..

[8] Veber D.F., Johnson S.R., Cheng H.Y., Smith B.R., Ward K.W., Kopple K.D. (2002). Molecular properties that influence the oral bioavailability of drug candidates. J. Med. Chem..

[9] Palucki M., Higgins J.D., Kwong E., Templeton A.C. (2010). Strategies at the interface of drug discovery and development: Early optimization of the solid state phase and preclinical toxicology formulation for potential drug candidates. J. Med. Chem..

[10] Ting J.M., Porter W.W., Mecca J.M., Bates F.S., Reineke T.M. (2018). Advances in polymer design for enhancing oral drug solubility and delivery. Bioconjug. Chem..

[11] Williams H.D., Trevaskis N.L., Charman S.A., Shanker R.M., Charman W.N., Pouton C.W., Porter C.J. (2013). Strategies to address low drug solubility in discovery and development. Pharm. Rev..

[12] Rasenack N., Muller B.W. (2004). Micron-size drug particles: Common and novel micronization techniques. Pharm. Dev. Technol..

[13] Zhang F., Aaltonen J., Tian F., Saville D.J., Rades T. (2009). Influence of particle size and preparation methods on the physical and chemical stability of amorphous simvastatin. Eur. J. Pharm. Biopharm..

[14] Elder D.P., Holm R., Diego H.L. (2013). Use of pharmaceutical salts and cocrystals to address the issue of poor solubility. Int. J. Pharm..

[15] Serajuddin A.T. (2007). Salt formation to improve drug solubility. Adv. Drug Deliv. Rev..

[16] Kalepu S., Manthina M., Padavala V. (2013). Oral lipid-based drug delivery systems–An overview. Acta Pharm. Sin. B.

[17] Shrestha H., Bala R., Arora S. (2014). Lipid-Based drug delivery systems. J. Pharm..

[18] Saokham P., Muankaew C., Jansook P., Loftsson T. (2018). Solubility of cyclodextrins and drug/cyclodextrin complexes. Molecules.

[19] Baghel S., Cathcart H., O’Reilly N.J. (2016). Polymeric amorphous solid dispersions: A review of amorphization, crystallization, stabilization, solid-state characterization, and aqueous solubilization of biopharmaceutical classification system class II drugs. J. Pharm. Sci..

[20] Huang Y., Dai W.G. (2014). Fundamental aspects of solid dispersion technology for poorly soluble drugs. Acta Pharm. Sin. B.

[21] Vasconcelos T., Sarmento B., Costa P. (2007). Solid dispersions as strategy to improve oral bioavailability of poor water soluble drugs. Drug Discov. Today.

[22] Vo C.L., Park C., Lee B.J. (2013). Current trends and future perspectives of solid dispersions containing poorly water-soluble drugs. Eur. J. Pharm. Biopharm..

[23] Janssens S., Van den Mooter G. (2009). Review: Physical chemistry of solid dispersions. J. Pharm. Pharm..

[24] Wegiel L.A., Mauer L.J., Edgar K.J., Taylor L.S. (2013). Crystallization of amorphous solid dispersions of resveratrol during preparation and storage-Impact of different polymers. J. Pharm. Sci..

[25] Van Zee N.J., Hillmyer M.A., Lodge T.P. (2020). Role of polymer excipients in the kinetic stabilization of drug-rich nanoparticles. ACS Appl. Bio Mater..

[26] Ma X., Williams R.O. (2019). Characterization of amorphous solid dispersions: An update. J. Drug Deliv. Sci. Technol..

[27] Ricarte R.G., Van Zee N.J., Li Z., Johnson L.M., Lodge T.P., Hillmyer M.A. (2019). Recent advances in understanding the micro-and nanoscale phenomena of amorphous solid dispersions. Mol. Pharm..

[28] Shi Q., Li F., Yeh S., Wang Y., Xin J. (2020). Physical stability of amorphous pharmaceutical solids: Nucleation, crystal growth, phase separation and effects of the polymers. Int. J. Pharm..

[29] Yu L. (2001). Amorphous pharmaceutical solids: Preparation, characterization and stabilization. Adv. Drug Deliv. Rev..

[30] Yang F., Su Y., Small J., Huang C., Martin G.E., Farrington A.M., DiNunzio J., Brown C.D. (2020). Probing the molecular-level interactions in an active pharmaceutical ingredient (API)–Polymer dispersion and the resulting impact on drug product formulation. Pharm. Res..

[31] Tran P., Pyo Y.C., Kim D.H., Lee S.E., Kim J.K., Park J.S. (2019). Overview of the manufacturing methods of solid dispersion technology for improving the solubility of poorly water-soluble drugs and application to anticancer drugs. Pharmaceutics.

[32] Zhang J., Han R., Chen W., Zhang W., Li Y., Ji Y., Chen L., Pan H., Yang X., Pan W. (2018). Analysis of the literature and patents on solid dispersions from 1980 to 2015. Molecules.

[33] Ahmad S., Johnston B.F., Mackay S.P., Schatzlein A.G., Gellert P., Sengupta D., Uchegbu I.F. (2010). In silico modelling of drug-polymer interactions for pharmaceutical formulations. J. R. Soc. Interface.

[34] Hossain S., Kabedev A., Parrow A., Bergstrom C.A.S., Larsson P. (2019). Molecular simulation as a computational pharmaceutics tool to predict drug solubility, solubilization processes and partitioning. Eur. J. Pharm. Biopharm..

[35] Das T., Mehta C.H., Nayak U.Y. (2020). Multiple approaches for achieving drug solubility: An in silico perspective. Drug Discov. Today.

[36] DeBoyace K., Wildfong P.L.D. (2018). The application of modeling and prediction to the formation and stability of amorphous solid dispersions. J. Pharm. Sci..

[37] Greenhalgh D.J., Williams A.C., Timmins P., York P. (1999). Solubility parameters as predictors of miscibility in solid dispersions. J. Pharm. Sci..

[38] Hancock B.C., York P., Rowe R.C. (1997). The use of solubility parameters in pharmaceutical dosage form design. Int. J. Pharm..

[39] Mollet H., Grubenmann A. (2000). Chapter 8: Solubility parameters, log, P., LSER, M numbers. Formulation Technology: Emulsions, Suspensions, Solid Forms.

[40] Li C., Strachan A. (2018). Cohesive energy density and solubility parameter evolution during the curing of thermoset. Polymer.

[41] Ahmad H., Yaseen M. (1979). Application of a chemical group contribution technique for calculating solubility parameters of polymers. Polym. Eng. Sci..

[42] Fedors R. (1974). A method for estimating both the solubility parameters and molar volumes of liquids. Polym. Eng. Sci..

[43] Krevelen-Van D.W., Hoftyzer P.J. (1976). Their Correlation with Chemical Structure, Their Numerical Estimation and Prediction from Additive Group Contributions.

[44] Telang C., Mujumdar S., Mathew M. (2009). Improved physical stability of amorphous state through acid base interactions. J. Pharm. Sci..

[45] Frisch M.J., Trucks G.W., Schlegel H.B., Scuseria G.E., Robb M.A., Cheeseman J.R., Scalmani G., Barone V., Petersson G.A., Nakatsuji H. (2016). Gaussian 16 Rev. C.01.

[46] Maniruzzaman M., Morgan D.J., Mendham A.P., Pang J., Snowden M.J., Douroumis D. (2013). Drug-polymer intermolecular interactions in hot-melt extruded solid dispersions. Int. J. Pharm..

[47] Maniruzzaman M., Pang J., Morgan D.J., Douroumis D. (2015). Molecular modeling as a predictive tool for the development of solid dispersions. Mol. Pharm..

[48] Nie H., Mo H., Zhang M., Song Y., Fang K., Taylor L.S., Li T., Byrn S.R. (2015). Investigating the interaction pattern and structural elements of a drug-polymer complex at the molecular level. Mol. Pharm..

[49] Trott O., Olson A.J. (2010). AutoDock Vina: Improving the speed and accuracy of docking with a new scoring function, efficient optimization, and multithreading. J. Comput. Chem..

[50] Barmpalexis P., Karagianni A., Katopodis K., Vardaka E., Kachrimanis K. (2019). Molecular modelling and simulation of fusion-based amorphous drug dispersions in polymer/plasticizer blends. Eur. J. Pharm. Sci..

[51] Shenogin S., Ozisik R. (2004). Xenoview: Visualization for Atomistic Simulations. http://www.vemmer.org/xenoview/xenoview.html.

[52] Kapourani A., Chatzitheodoridou M., Kontogiannopoulos K.N., Barmpalexis P. (2020). Experimental, thermodynamic, and molecular modeling evaluation of amorphous simvastatin-poly(vinylpyrrolidone) solid dispersions. Mol. Pharm..

[53] Froimowitz M. (1993). HyperChem: A software package for computational chemistry and molecular modeling. Biotechniques.

[54] Mosquera-Giraldo L.I., Borca C.H., Meng X., Edgar K.J., Slipchenko L.V., Taylor L.S. (2016). Mechanistic design of chemically diverse polymers with applications in oral drug delivery. Biomacromolecules.

[55] (2020). Biovia. Biovia Materials Studio: An Integrated, Multi-Scale Modeling Environment. https://www.3ds.com/products-services/biovia/products/molecular-modeling-simulation/biovia-materials-studio/.

[56] Erlebach A., Ott T., Otzen C., Schubert S., Czaplewska J., Schubert U.S., Sierka M. (2016). Thermodynamic compatibility of actives encapsulated into PEG-PLA nanoparticles: In Silico predictions and experimental verification. J. Comput. Chem..

[57] Gupta J., Nunes C., Vyas S., Jonnalagadda S. (2011). Prediction of solubility parameters and miscibility of pharmaceutical compounds by molecular dynamics simulations. J. Phys. Chem. B.

[58] Iesavand H., Rahmati M., Afzali D., Modiri S. (2019). Investigation on absorption and release of mercaptopurine anticancer drug from modified polylactic acid as polymer carrier by molecular dynamic simulation. Mater. Sci. Eng. C Mater. Biol. Appl..

[59] Jha P.K., Larson R.G. (2014). Assessing the efficiency of polymeric excipients by atomistic molecular dynamics simulations. Mol. Pharm..

[60] Macháčková M., Tokarsky J., Capkova P. (2013). A simple molecular modeling method for the characterization of polymeric drug carriers. Eur. J. Pharm. Sci..

[61] Razmimanesh F., Amjad-Iranagh S., Modarress H. (2015). Molecular dynamics simulation study of chitosan and gemcitabine as a drug delivery system. J. Mol. Model..

[62] Shahzad Y., Sohail S., Arshad M.S., Hussain T., Shah S.N. (2013). Development of solid dispersions of artemisinin for transdermal delivery. Int. J. Pharm..

[63] Yani Y., Kanaujia P., Chow P.S., Tan R.B.H. (2017). Effect of API-Polymer miscibility and interaction on the stabilization of amorphous solid dispersion: A molecular simulation study. Ind. Eng. Chem. Res..

[64] Case D.A., Cheatham T.E., Darden T., Gohlke H., Luo R., Merz K.M., Onufriev A., Simmerling C., Wang B., Woods R.J. (2005). The Amber biomolecular simulation programs. J. Comput. Chem..

[65] Salomon-Ferrer R., Case D.A., Walker R.C. (2013). An overview of the Amber biomolecular simulation package. Wires Comput. Mol. Sci..

[66] Chan T., Ouyang D. (2018). Investigating the molecular dissolution process of binary solid dispersions by molecular dynamics simulations. Asian J. Pharm. Sci..

[67] Han R., Huang T., Liu X., Yin X., Li H., Lu J., Ji Y., Sun H., Ouyang D. (2019). Insight into the dissolution molecular mechanism of ternary solid dispersions by combined experiments and molecular simulations. AAPS Pharmscitech.

[68] Xiang T.X., Anderson B.D. (2013). Molecular dynamics simulation of amorphous indomethacin-poly(vinylpyrrolidone) glasses: Solubility and hydrogen bonding interactions. J. Pharm. Sci..

[69] Xiang T.X., Anderson B.D. (2017). Molecular dynamics simulation of amorphous hydroxypropylmethylcellulose and its mixtures with felodipine and water. J. Pharm. Sci..

[70] Van Der Spoel D., Lindahl E., Hess B., Groenhof G., Mark A.E., Berendsen H.J. (2005). GROMACS: Fast, flexible, and free. J. Comput. Chem..

[71] Hess B., Kutzner C., van der Spoel D., Lindahl E. (2008). GROMACS 4: Algorithms for highly efficient, load-balanced, and scalable molecular simulation. J. Chem. Theory Comput..

[72] Brunsteiner M., Khinast J., Paudel A. (2018). Relative contributions of solubility and mobility to the stability of amorphous solid dispersions of poorly soluble drugs: A molecular dynamics simulation study. Pharmaceutics.

[73] Plimpton S. (1995). Fast parallel algorithms for short-range molecular dynamics. J. Comput. Phys..

[74] Eslami M., Nikkhah S.J., Hashemianzadeh S.M., Sajadi S.A. (2016). The compatibility of Tacrine molecule with poly(n-butylcyanoacrylate) and Chitosan as efficient carriers for drug delivery: A molecular dynamics study. Eur. J. Pharm. Sci..

[75] Phillips J.C., Braun R., Wang W., Gumbart J., Tajkhorshid E., Villa E., Chipot C., Skeel R.D., Kalé L., Schulten K. (2005). Scalable molecular dynamics with NAMD. J. Comput. Chem..

[76] Gao Y., Olsen K.W. (2015). Drug-polymer interactions at water-crystal interfaces and implications for crystallization inhibition: Molecular dynamics simulations of amphiphilic block copolymer interactions with tolazamide crystals. J. Pharm. Sci..

[77] (2020). Schrödinger Release 2020-4: Maestro.

[78] Fule R., Meer T., Sav A., Amin P. (2013). Solubility and dissolution rate enhancement of lumefantrine using hot melt extrusion technology with physicochemical characterisation. J. Pharm. Investig..

[79] Gangurde A.B., Kundaikar H.S., Javeer S.D., Jaiswar D.R., Degani M.S., Amin P.D. (2015). Enhanced solubility and dissolution of curcumin by a hydrophilic polymer solid dispersion and its insilico molecular modeling studies. J. Drug Deliv. Sci. Technol..

[80] (2020). Schrödinger Release 2020-4: Desmond Molecular Dynamics System, D. E. Shaw Research, New York, NY, 2020.

[81] Jadhav P., Gokarna V., Deshpande V., Vavia P. (2020). bioavailability enhancement of olmesartan medoxomil using hot-melt extrusion: In-silico, in-vitro, and in-vivo evaluation. AAPS Pharmscitech.

[82] Mazurek A.H., Szeleszczuk L., Pisklak D.M. (2020). Periodic DFT calculations-review of applications in the pharmaceutical sciences. Pharmaceutics.

[83] Andreoni W., Curioni A., Mordasini T. (2001). DFT-based molecular dynamics as a new tool for computational biology: First applications and perspective. IBM J. Res. Dev..

[84] Car R., Parrinello M. (1985). Unified approach for molecular dynamics and density-functional theory. Phys. Rev. Lett..

[85] Mazurek A., Szeleszczuk L., Pisklak D.M. (2020). Can we predict the pressure induced phase transition of urea? Application of quantum molecular dynamics. Molecules.

[86] Van der Kamp M.W., Mulholland A.J. (2013). Combined quantum mechanics/molecular mechanics (QM/MM) methods in computational enzymology. Biochemistry.

[87] Meng F., Trivino A., Prasad D., Chauhan H. (2015). Investigation and correlation of drug polymer miscibility and molecular interactions by various approaches for the preparation of amorphous solid dispersions. Eur. J. Pharm. Sci..

[88] Wang B., Wang D., Zhao S., Huang X., Zhang J., Lv Y., Liu X., Lv G., Ma X. (2017). Evaluate the ability of PVP to inhibit crystallization of amorphous solid dispersions by density functional theory and experimental verify. Eur. J. Pharm. Sci..

[89] Bookwala M., DeBoyace K., Buckner I.S., Wildfong P.L.D. (2020). Predicting density of amorphous solid materials using molecular dynamics simulation. AAPS Pharmscitech.

[90] Xiang T.X., Anderson B.D. (2013). Molecular dynamics simulation of amorphous indomethacin. Mol. Pharm..

[91] Kasimova A.O., Pavan G.M., Danani A., Mondon K., Cristiani A., Scapozza L., Gurny R., Moller M. (2012). Validation of a novel molecular dynamics simulation approach for lipophilic drug incorporation into polymer micelles. J. Phys. Chem. B.

[92] Chen S., Li J., Wei L., Jin Y., Khosla T., Xiao J., Cheng B., Duan H. (2018). A molecular modeling study for miscibility of polyimide/polythene mixing systems with/without compatibilizer. J. Polym. Eng..

[93] Andrews A., Handler R.A., Blaisten-Barojas E. (2020). Structure, energetics and thermodynamics of PLGA condensed phases from Molecular Dynamics. Polymer.

[94] Muljajew I., Erlebach A., Weber C., Buchheim J.R., Sierka M., Schubert U.S. (2020). A polyesteramide library from dicarboxylic acids and 2,2′-bis(2-oxazoline): Synthesis, characterization, nanoparticle formulation and molecular dynamics simulations. Polym. Chem..

[95] Xiang T.X., Anderson B.D. (2014). Molecular dynamics simulation of amorphous hydroxypropyl-methylcellulose acetate succinate (HPMCAS): Polymer model development, water distribution, and plasticization. Mol. Pharm..

[96] Adhikari U., Goliaei A., Tsereteli L., Berkowitz M.L. (2016). Properties of poloxamer molecules and poloxamer micelles dissolved in water and next to lipid bilayers: Results from computer simulations. J. Phys. Chem. B.

[97] Gupta J., Nunes C., Jonnalagadda S. (2013). A molecular dynamics approach for predicting the glass transition temperature and plasticization effect in amorphous pharmaceuticals. Mol. Pharm..

[98] Ma S.M., Zhao L., Wang Y.L., Zhu Y.L., Lu Z.Y. (2020). The coarse-grained models of poly(ethylene oxide) and poly(propylene oxide) homopolymers and poloxamers in big multipole water (BMW) and MARTINI frameworks. Phys. Chem. Chem. Phys..

[99] Rigby D., Sun H., Eichinger B.E. (1997). Computer simulations of poly(ethylene oxide): Force field, pvt diagram and cyclization behaviour. Polym. Int..

[100] Sun H. (1998). COMPASS:  An ab initio force-field optimized for condensed-phase applicationsoverview with details on alkane and benzene compounds. J. Phys. Chem. B.

[101] Maple J.R., Dinur U., Hagler A.T. (1988). Derivation of force fields for molecular mechanics and dynamics from ab initio energy surfaces. Proc. Natl. Acad. Sci. USA.

[102] Brooks B.R., Bruccoleri R.E., Olafson B.D., States D.J., Swaminathan S., Karplus M. (1983). CHARMM: A program for macromolecular energy, minimization, and dynamics calculations. J. Comput. Chem..

[103] MacKerell A.D., Banavali N., Foloppe N. (2000). Development and current status of the CHARMM force field for nucleic acids. Biopolymers.

[104] Wang J., Wang W., Kollman P.A., Case D.A. (2006). Automatic atom type and bond type perception in molecular mechanical calculations. J. Mol. Graph. Model..

[105] Wang J., Wolf R.M., Caldwell J.W., Kollman P.A., Case D.A. (2004). Development and testing of a general amber force field. J. Comput. Chem..

[106] Jorgensen W.L., Maxwell D.S., Tirado-Rives J. (1996). Development and testing of the OPLS all-atom force field on conformational energetics and properties of organic liquids. J. Am. Chem. Soc..

[107] Bhattacharya S., Suryanarayanan R. (2009). Local mobility in amorphous pharmaceuticals–Characterization and implications on stability. J. Pharm. Sci..

[108] Kothari K., Ragoonanan V., Suryanarayanan R. (2014). Influence of molecular mobility on the physical stability of amorphous pharmaceuticals in the supercooled and glassy States. Mol. Pharm..

[109] Pajula K., Taskinen M., Lehto V.P., Ketolainen J., Korhonen O. (2010). Predicting the formation and stability of amorphous small molecule binary mixtures from computationally determined Flory-Huggins interaction parameter and phase diagram. Mol. Pharm..

[110] Turpin E.R., Taresco V., Al-Hachami W.A., Booth J., Treacher K., Tomasi S., Alexander C., Burley J., Laughton C.A., Garnett M.C. (2018). In silico screening for solid dispersions: The trouble with solubility parameters and chiFH. Mol. Pharm..

[111] Paus R., Prudic A., Ji Y. (2015). Influence of excipients on solubility and dissolution of pharmaceuticals. Int. J. Pharm..

[112] Craig M., Duncan Q. (2002). The mechanisms of drug release from solid dispersions in water-soluble polymers. Int. J. Pharm..

[113] Puncochova K., Ewing A.V., Gajdosova M., Sarvasova N., Kazarian S.G., Beranek J., Stepanek F. (2015). Identifying the mechanisms of drug release from amorphous solid dispersions using MRI and ATR-FTIR spectroscopic imaging. Int. J. Pharm..

[114] Xie T., Gao W., Taylor L.S. (2017). Impact of Eudragit EPO and hydroxypropyl methylcellulose on drug release rate, supersaturation, precipitation outcome and redissolution rate of indomethacin amorphous solid dispersions. Int. J. Pharm..

[115] Bhugra C., Pikal M.J. (2008). Role of thermodynamic, molecular, and kinetic factors in crystallization from the amorphous state. J. Pharm. Sci..

[116] Kalinin A.A., Higgins G.A., Reamaroon N., Soroushmehr S., Allyn-Feuer A., Dinov I.D., Najarian K., Athey B.D. (2018). Deep learning in pharmacogenomics: From gene regulation to patient stratification. Pharmacogenomics.

[117] Kastrin A., Ferk P., Leskosek B. (2018). Predicting potential drug-drug interactions on topological and semantic similarity features using statistical learning. PLoS ONE.

[118] Zuvela P., David J., Wong M.W. (2018). Interpretation of ANN-based QSAR models for prediction of antioxidant activity of flavonoids. J. Comput. Chem..

[119] Alberi K., Nardelli M.B., Zakutayev A., Mitas L., Curtarolo S., Jain A., Fornari M., Marzari N., Takeuchi I., Green M.L. (2019). The 2019 materials by design roadmap. J. Phys. D: Appl. Phys..

[120] Goldman B.B., Walters W.P., Spellmeyer D.C. (2006). Chapter 8. Machine learning in computational chemistry. Annual Reports in Computational Chemistry.

[121] Mendyk A., Szlȩk J., Jachowicz R., Aguilar J.E. (2013). 3-ME_expert 2.0: A heuristic decision support system for microemulsions formulation development. Formulation Tools for Pharmaceutical Development.

[122] Rowe R.C., Roberts R.J. (1998). Artificial intelligence in pharmaceutical product formulation: Knowledge-based and expert systems. Pharm. Sci. Technol. Today.

[123] Zhang Z.-H., Pan W.-S., Aguilar J.E. (2013). 4–Expert system for the development and formulation of push–pull osmotic pump tablets containing poorly water-soluble drugs. Formulation Tools for Pharmaceutical Development.

[124] Gubaev K., Podryabinkin E.V., Hart G.L.W., Shapeev A.V. (2019). Accelerating high-throughput searches for new alloys with active learning of interatomic potentials. Comput. Mater. Sci..

[125] Hase F., Roch L.M., Kreisbeck C., Aspuru-Guzik A. (2018). Phoenics: A Bayesian optimizer for chemistry. ACS Cent. Sci..

[126] Tran K., Ulissi Z.W. (2018). Active learning across intermetallics to guide discovery of electrocatalysts for CO2 reduction and H2 evolution. Nat. Catal..

[127] Schmidhuber J. (2015). Deep learning in neural networks: An overview. Neural Netw..

[128] Das M., Chakraborty T. (2016). ANN in Pharmaceutical Product and Process Development. Artificial Neural Network for Drug Design, Delivery and Disposition.

[129] Han R., Yang Y., Li X., Ouyang D. (2018). Predicting oral disintegrating tablet formulations by neural network techniques. Asian J. Pharm. Sci..

[130] Han R., Xiong H., Ye Z., Yang Y., Huang T., Jing Q., Lu J., Pan H., Ren F., Ouyang D. (2019). Predicting physical stability of solid dispersions by machine learning techniques. J. Control. Release.

[131] Katoch S., Chauhan S.S., Kumar V. (2020). A review on genetic algorithm: Past, present, and future. Multimed. Tools Appl..

[132] Boateng E.Y., Abaye D.A. (2019). A review of the logistic regression model with emphasis on medical research. J. Data Anal. Inf. Process..

[133] Kotsiantis S.B. (2013). Decision trees: A recent overview. Artif. Intell. Rev..

[134] Fawagreh K., Gaber M.M., Elyan E. (2014). Random forests: From early developments to recent advancements. Syst. Sci. Control. Eng..

[135] Zhang Z. (2016). Introduction to machine learning: K-nearest neighbors. Ann. Transl. Med..

[136] Rajiv S., Sourish D., Sujit K.S. (2020). A Bayesian perspective of statistical machine learning for big data. Comput. Stat..

[137] Natekin A., Knoll A. (2013). Gradient boosting machines, a tutorial. Front. Neurorobot..

[138] Barmpalexis P., Kachrimanis K., Georgarakis E. (2011). Solid dispersions in the development of a nimodipine floating tablet formulation and optimization by artificial neural networks and genetic programming. Eur. J. Pharm. Biopharm..

[139] Moore M.D., Wildfong P.L. (2011). Informatics calibration of a molecular descriptors database to predict solid dispersion potential of small molecule organic solids. Int. J. Pharm..

[140] Barmpalexis P., Koutsidis I., Karavas E., Louka D., Papadimitriou S.A., Bikiaris D.N. (2013). Development of PVP/PEG mixtures as appropriate carriers for the preparation of drug solid dispersions by melt mixing technique and optimization of dissolution using artificial neural networks. Eur. J. Pharm. Biopharm..

[141] Medarević D.P., Kleinebudde P., Djuris J., Djuric Z., Ibric S. (2016). Combined application of mixture experimental design and artificial neural networks in the solid dispersion development. Drug Dev. Ind. Pharm..

[142] Alhalaweh A., Alzghoul A., Kaialy W., Mahlin D., Bergstrom C.A. (2014). Computational predictions of glass-forming ability and crystallization tendency of drug molecules. Mol. Pharm..

[143] Nurzyńska K., Booth J., Roberts C.J., McCabe J., Dryden I., Fischer P.M. (2015). Long-Term amorphous drug stability predictions using easily calculated, predicted, and measured parameters. Mol. Pharm..

[144] Przybyłek M., Jeliński T., Cysewski P. (2019). Application of multivariate adaptive regression splines (MARSplines) for predicting hansen solubility parameters based on 1D and 2D molecular descriptors computed from SMILES string. J. Chem..

[145] Shen L., Yang W. (2018). Molecular dynamics simulations with quantum mechanics/molecular mechanics and adaptive neural networks. J. Chem. Theory Comput..

